# Computational Investigation of the Therapeutic Potential of *Detarium senegalense* in the Management of Erectile Dysfunction

**DOI:** 10.3390/ijms252212362

**Published:** 2024-11-18

**Authors:** Jerius Nkwuda Ejeje, Emmanuel Ayodeji Agbebi, Makhosazana Siduduzile Mathenjwa-Goqo, Obinna Aru Oje, Precious Eseose Agboinghale, Ikechukwu Theophilus Ebe, Tajudeen Olabisi Obafemi, Ezekiel Adewole, Omaka N. Omaka, Sunday Amos Onikanni, Basiru Olaitan Ajiboye, Olaposi Idowu Omotuyi, Babatunji Emmanuel Oyinloye

**Affiliations:** 1 Phytomedicine, Biochemical Toxicology and Biotechnology Research Laboratories, Department of Biochemistry, College of Sciences, Afe Babalola University, PMB 5454, Ado-Ekiti 360001, Nigeria; ejeje.jerius@funai.edu.ng (J.N.E.); preciousagboinghale@gmail.com (P.E.A.); oobafemi@abuad.edu.ng (T.O.O.); onikannisa@abuad.edu.ng (S.A.O.); 2 Department of Biochemistry, Faculty of Biological Sciences, Alex Ekwueme Federal University Ndufu Alike Ikwo, P.O. Box 1010, Abakaliki 480101, Nigeria; obinna.oje@funai.edu.ng; 3 Institute of Drug Research and Development, S.E. Bogoro Center, Afe Babalola University, PMB 5454, Ado-Ekiti 360001, Nigeria; agbebiea@abuad.edu.ng (E.A.A.); olaposi.omotuyi@abuad.edu.ng (O.I.O.); 4 Department of Pharmacognosy and Natural Products, College of Pharmacy, Afe Babalola University, Ado-Ekiti 360001, Nigeria; 5 Biotechnology and Structural Biology (BSB) Group, Department of Biochemistry and Microbiology, University of Zululand, KwaDlangezwa 3886, South Africa; mathenjwam@unizulu.ac.za; 6 Department of Chemistry and Biomolecular Sciences, Faculty of Science, University of Ottawa, Ottawa, ON K1N 6N5, Canada; 7 Department of Medical Biochemistry, Faculty of Basic Medical Sciences, David Umahi Federal University of Health Sciences, Uburu 491105, Nigeria; ikechukwuebe1@gmail.com; 8 Department of Chemical Sciences, College of Sciences, Afe Babalola University, PMB 5454, Ado-Ekiti 360001, Nigeria; adewolee@abuad.edu.ng; 9 Department of Chemistry, Faculty of Physical Sciences, Alex Ekwueme Federal University Ndufu Alike Ikwo, P.O. Box 1010, Abakaliki 480101, Nigeria; omaka.omaka@funai.edu.ng; 10 Phytomedicine and Molecular Toxicology Research Laboratory, Department of Biochemistry, Federal University Oye-Ekiti, Oye-Ekiti 360101, Nigeria; basiru.ajiboye@fuoye.edu.ng; 11 Department of Pharmacology and Toxicology, College of Pharmacy, Afe Babalola University, PMB 5454, Ado-Ekiti 360001, Nigeria

**Keywords:** erectile dysfunction, *Detarium senegalense*, phosphodiesterase 5, sildenafil, catechin, epicatechin

## Abstract

Erectile dysfunction (ED) is a multifactorial social problem affecting men worldwide. While phosphodiesterase type 5 inhibitors (PDE5) like sildenafil are commonly used, they often present side effects, underscoring the need for alternative therapies. Therefore, this study investigated the potential of phytochemicals from *Detarium senegalense* in the management of ED. A library of phytochemicals from *Detarium senegalense* was generated, prepared, and interacted with six key enzymes implicated in ED, including PDE5, using the Schrödinger Maestro suite. The results identified catechin, epicatechin, and gallic acid as the leading compounds with significant binding affinities for the targeted enzymes. Catechin and epicatechin (−9.877 and −11.408 kcal/mol, respectively) exhibited comparable binding affinities to sildenafil (−11.926 kcal/mol) on PDE5. The MD simulation results also revealed superior stability and ability to maintain interaction with key amino acids at the active site of PDE5 over the entire simulation period for these compounds. These compounds also demonstrated favorable ADMET profiles over sildenafil, including high gastrointestinal absorption and no violation of Lipinski’s rule, indicating good bioavailability and drug likeness. These findings suggest that flavonoids from *Detarium senegalense*, especially catechin and epicatechin, have potential in the management of ED by interacting with multiple targets involved in its pathogenesis.

## 1. Introduction

Erectile dysfunction (ED) is a multifactorial condition that is characterized by a persistent inability to achieve or sustain penile erection sufficiently for satisfactory sexual intercourse despite the presence of proper erotic stimuli [1,2,3]. It was previously/synonymously referred to as impotence [1,4]. Though this condition is usually common in men aged ≥40 years, with the prevalence increasing with increasing age and co-morbidities (e.g., diabetes and other cardiometabolic disorders), it is a social problem that can affect men of all ages, cultures, and ethnic groups, with a significant effect on the quality of life of the person and their partners [1]. Its prevalence has been estimated to be between 14% and 48% [1]. Specifically, the 2021 National Survey of Sexual Wellbeing estimated the prevalence of erectile dysfunction in the United States as 24.2%, with the prevalence increasing with age. However, this value could have been underestimated because of male reservedness and privacy [1,5,6]. It has been known to negatively impact the quality of life and can lead to loss of self-esteem, anxiety, and depression [7,8]. The etiology of ED is multifactorial, with aging, psychological, and neurological disorders and other co-morbidities/organic causes like cardiovascular and metabolic disorders the key contributing factors. Benign prostate hyperplasia (BPH) and some medications (antipsychotic, antidepressant, etc.) can also cause ED. The relaxation of the intracavernosal smooth muscle is the key critical process in penile erection [2,9]. It involves a series of process involving nitric oxide (NO), which stimulates cyclic guanosine monophosphate (cGMP) production, which in turn activates protein kinase G, closing the calcium channel. This causes the intracavernosal smooth muscle to relax and increases the arterial blood flow, resulting in a rigid erection. The degradation of the cGMP by penile phosphodiesterase (PDE) reverses this process by contracting the corporal smooth muscle. Therefore, any problem along these pathway/process can lead to erectile dysfunction [2,9,10,11]. Current treatments in the market target key players along this pathway to help in the management of ED. An example is sildenafil (Viagra^®^), a very commonly used drug. It is a phosphodiesterase inhibitor that prevents cGMP degradation by inhibiting penile PDE, thus leading to sustained erection. However, these drugs are bedeviled by serious side effects, including headache, dizziness, flushing, and cardiovascular complications [1,12,13]. Therefore, there is a need for new therapy with fewer side effects and comparable efficacy. The use of medicinal plants with aphrodisiac properties was the mainstay for treatment of ED in previous centuries [1,14]. Medicinal plants have made key contributions to the drug development process [15], and several medicinal plants have shown promising potential in the management of ED, including ginseng, *Lepidium meyenii*, *Pinus pinaster*, *Tribulus terrestris*, and saffron, among others [16]. The use of medicinal plants in the management of ED is gaining attention in underdeveloped countries, mainly because of the affordability and reported minimal side effects with the use of medicinal plants in complementary therapies [17,18,19]. Studies have shown that some phenolic compounds (particularly flavonoids) possess PDE5-inhibitory activity [20]. Moreover, *Garcinia kola* [21] and *Ocimum gratissimum* [22] have been studied and found to inhibit key markers of ED. Aside from PDE5 inhibitors, other viable therapeutic options should be considered for the management of ED. From various reports, adenosine deaminase, arginase, O-GlcNAc transferase (OGT), phosphodiesterase 5, protein kinase C (PKC), and steroidogenic cytochrome P450 17A1 have all been implicated in the etiology of erectile dysfunction [23,24]. The depletion of adenosine, L-arginine, OGT, testosterone; dihydrotestosterone (DHT), second messenger molecules (cAMP and cGMP), and nitric oxide (NO) occurs with the activation of these enzymes. Therefore, the inhibition of these enzymes will play a key role in the management of erectile dysfunction [23,25]. This study, therefore, aims to explore the potential of *Detarium senegalense* in the management of erectile dysfunction via its inhibitory potential on these enzymes.

**Figure 1:**
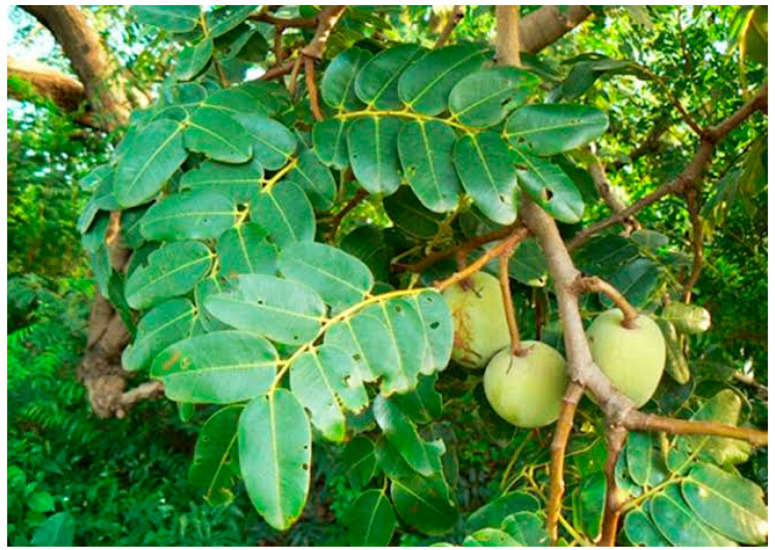
*Detarium senegalense* J.F.Gmel. in its natural habitat.

*Detarium senegalense* J.F.Gmel. (Figure 1) is a medicinal plant native to the west African region and is commonly known as the African star apple or tallow tree [26,27]. It is a member of the family Fabaceae, and various parts of the plant, including the bark, leaves, and fruits, have been used traditionally for culinary and medicinal purposes. Recent scientific investigations have unveiled the rich phytochemical composition of *Detarium senegalense*, which includes flavonoids, tannins, saponins, alkaloids, and phenolic compounds [26]. These bioactive constituents are endowed with diverse pharmacological properties, such as antidiabetic, antidiarrheal, antioxidant, anti-inflammatory, antimicrobial, and vasodilatory effects [26,28,29]. Since the pathophysiology of erectile dysfunction involves an intricate interplay among vascular, neural, hormonal, and psychological factors, several phytochemicals present in this plant exhibit promising pharmacological activities that may address the underlying mechanisms contributing to ED. Flavonoids, such as quercetin and kaempferol, possess antioxidant properties and can enhance endothelial function by promoting nitric oxide (NO) production and inhibiting oxidative stress-induced endothelial dysfunction [23,30]. Additionally, saponins found in the plant may modulate hormonal pathways implicated in erectile function, including the regulation of testosterone levels. The vasodilatory effects of the phytochemicals present in the plant are particularly relevant to erectile function, as adequate penile blood flow is indispensable for achieving and maintaining erection. Flavonoids exert vasodilatory effects through various mechanisms, including the upregulation of endothelial nitric oxide synthase (eNOS) expression and the inhibition of vasoconstrictor pathways. Furthermore, saponins may enhance cavernosal smooth muscle relaxation by modulating intracellular calcium levels and potassium channel activity [31]. This makes the plant a promising one and led to our investigation of its potential in the management of ED via its interaction with key enzymes involved in erectile dysfunction pathogenesis.

## 2. Results

**Figure 2:**
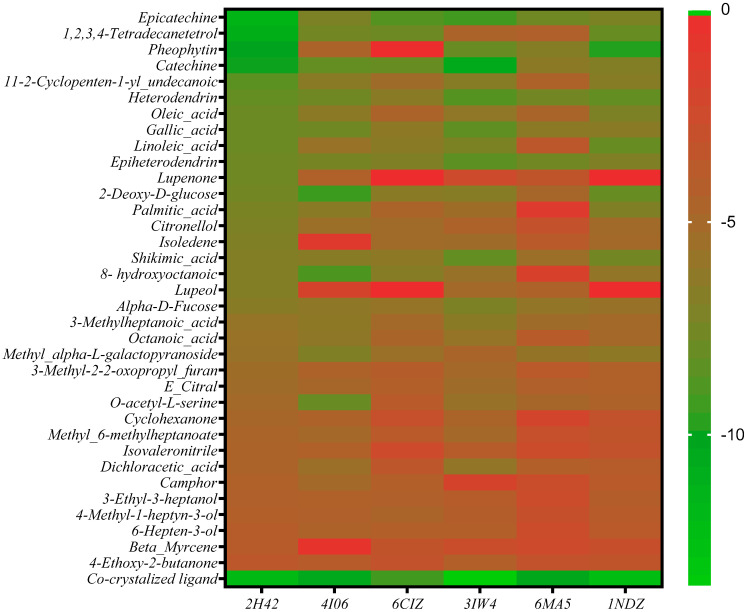
Heatmap showing the Glide scores of phytochemicals from *Detarium senegalense* against human PDE5, arginase 2, steroidogenic cytochrome P450 17A1, protein kinase C alpha, OGT, and adenosine deaminase.

Molecular docking was performed to generate the glide scores, which is predictive of the binding affinity of the ligands with each receptor and consequently their activity. The Glide scores of the ligands at each receptor’s active site are presented as a heatmap in Figure 2 below.

**Figure 3:**
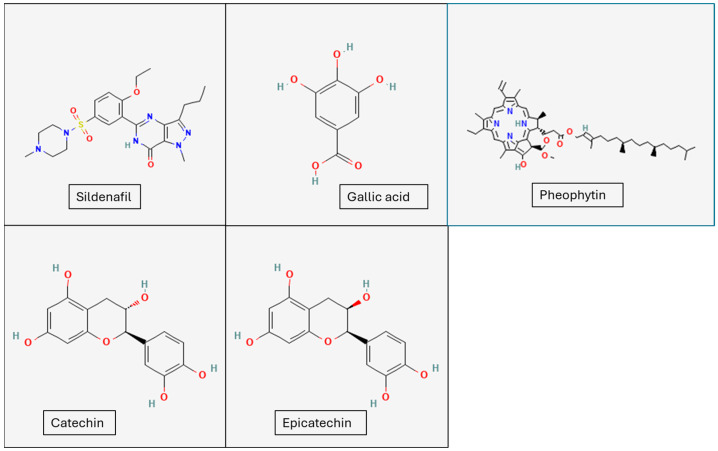
Structures of sildenafil, gallic acid, pheophytin, catechin, and epicatechin (source: PubChem online database https://pubchem.ncbi.nlm.nih.gov/).

From the results above, it was observed that catechin and its isomer, epicatechin; gallic acid, and pheophytin had good binding affinity with the receptors, and these were therefore selected as our hit compounds. The chemical structures of these hit compounds and sildenafil are provided in Figure 3. Across most of the receptors, catechin and epicatechin had better binding affinity than the other hit compounds and a comparable binding affinity to the co-crystallized ligands. For example, on the PDE5 enzyme, catechin and epicatechin had binding affinity of −9.877 and −11.408 kcal/mol compared with the −11.926 kcal/mol value of the co-crystallized ligand, sildenafil, a standard drug in the market for the treatment of erectile dysfunction. These compounds also exhibited similar binding affinity to the co-crystallized ligand at the active site of the steroidogenic cytochrome P450 17A1 enzyme with binding affinity of −7.921 and −8.547 kcal/mol, respectively, compared with the −9.029 kcal/mol value of the co-crystallized ligand. These values are presented in Table 1 below.

**Figure 4:**
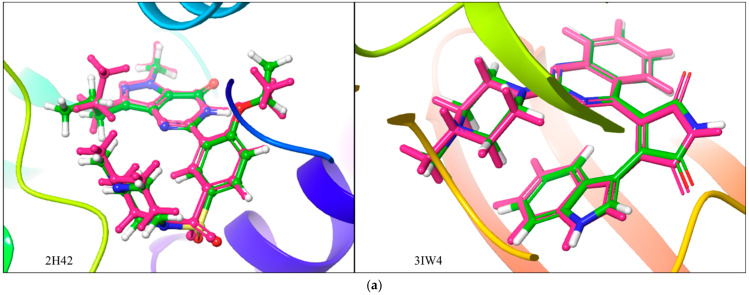

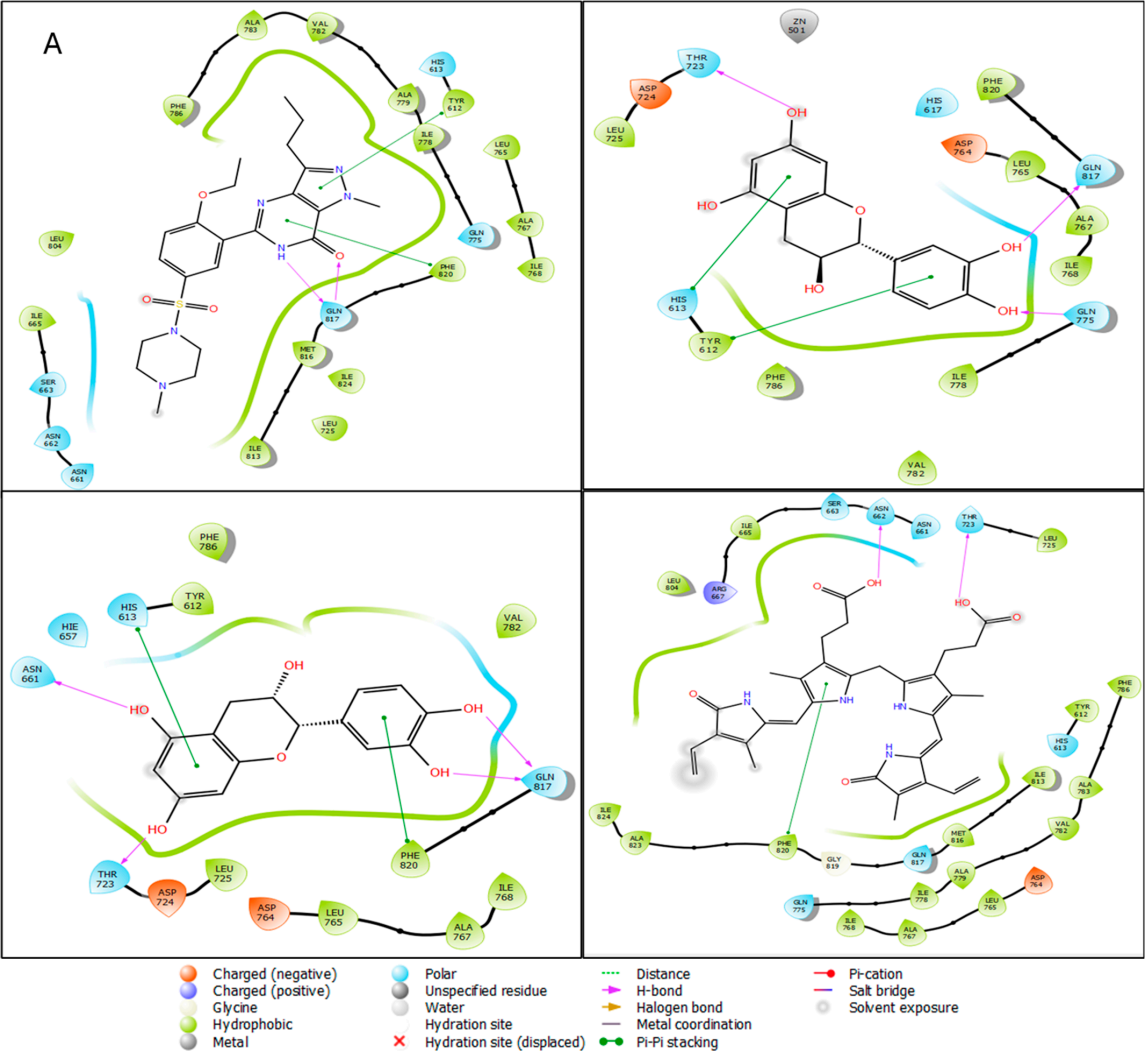

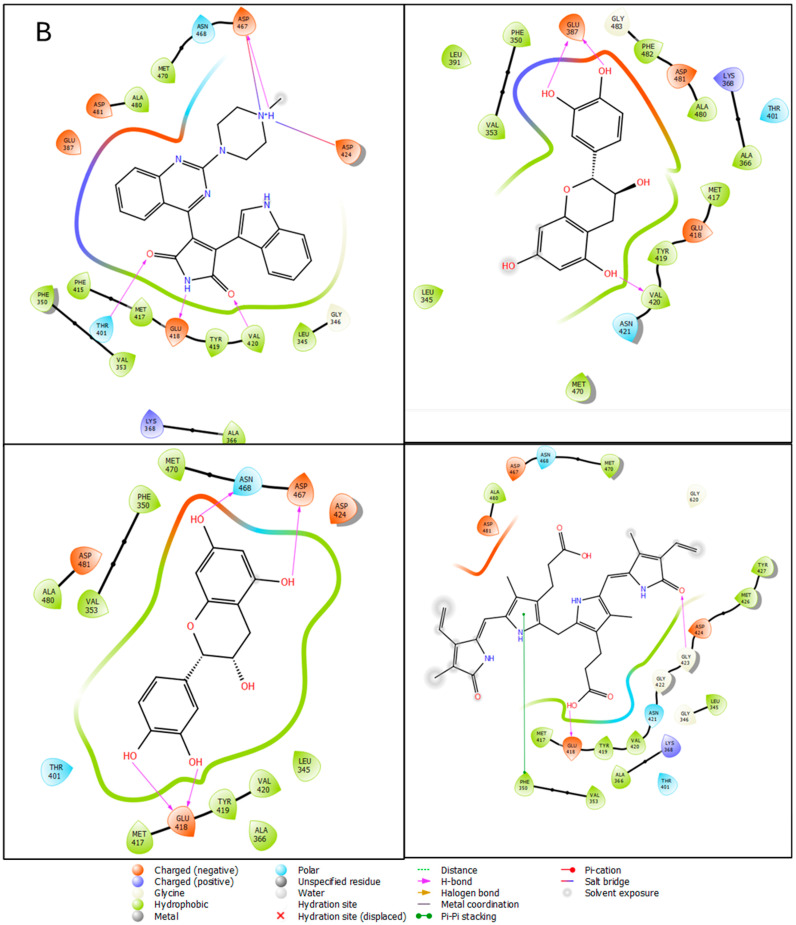

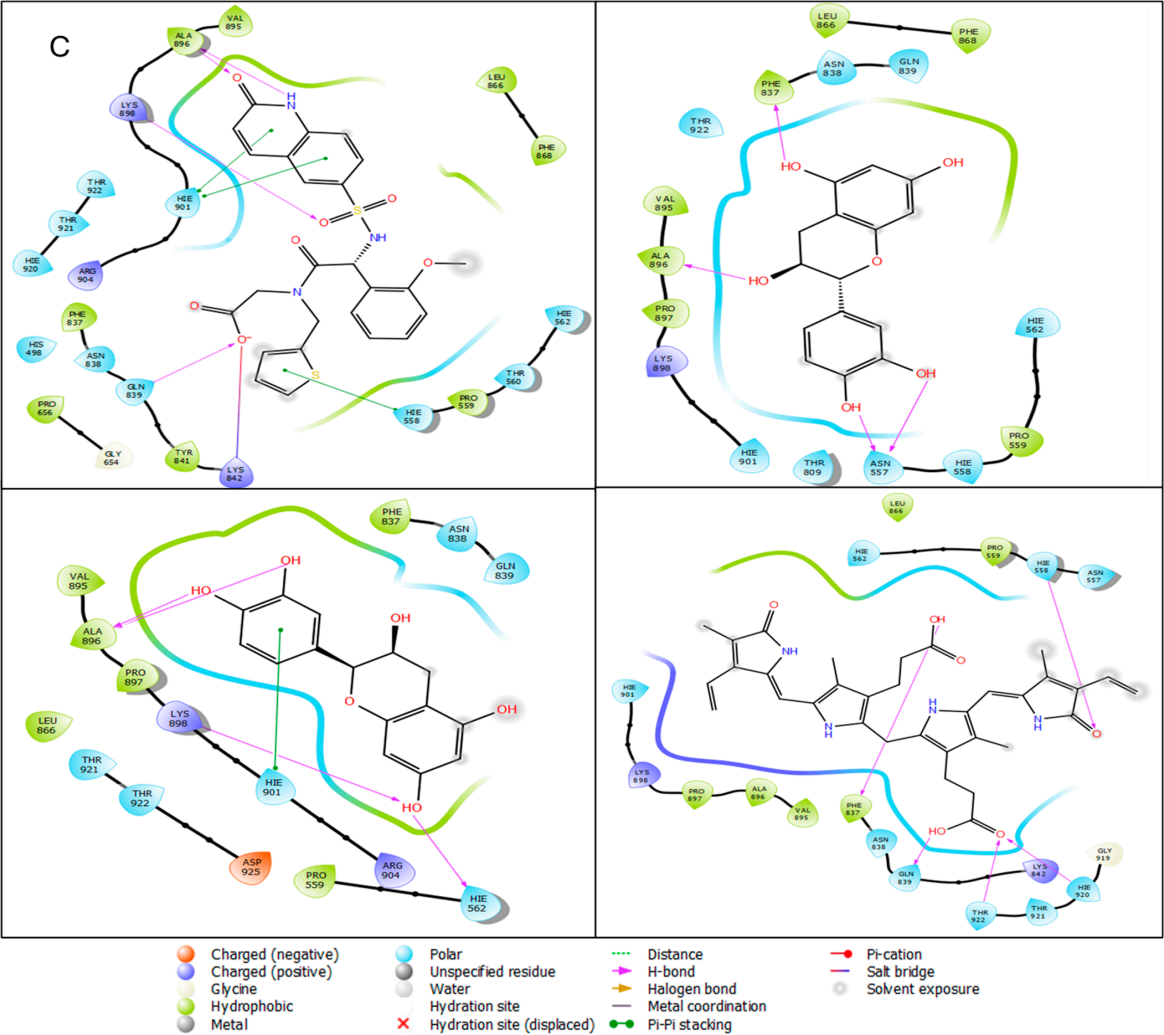

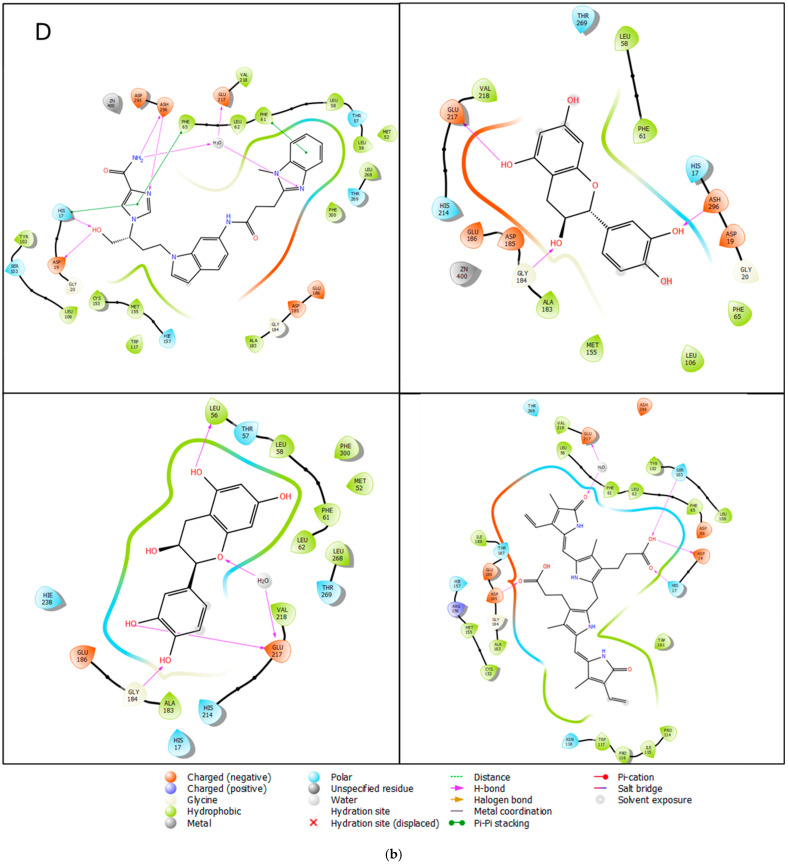

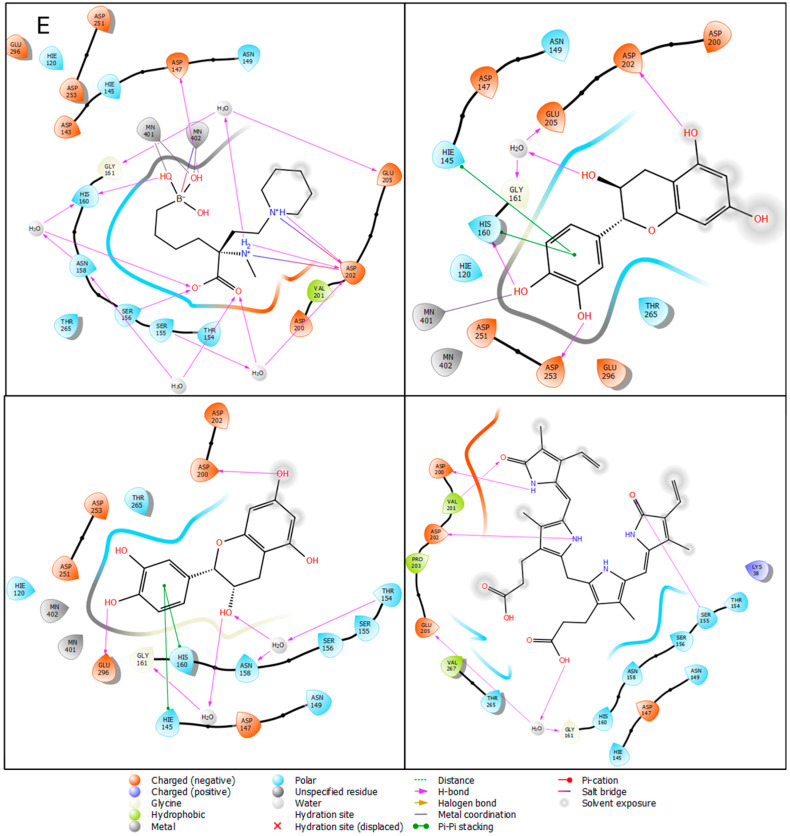

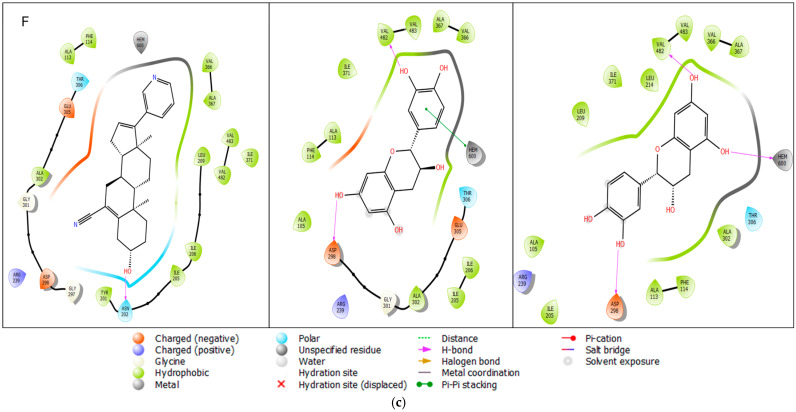
(**a**) Superimposed structures of the co-crystalized ligands in their co-crystallized (green) and re-docked poses (magenta) at the active sites of the receptors. (RMSD = 0.65 and 0.26 A for 2H42 and 3IW4, respectively). (**b**) 2D ligand interactions of the co-crystallized ligand (CCL), catechin, epicatechin, and pheophytin with (**A**) human PDE5, (**B**) protein kinase C alpha, (**C**) OGT, and (**D**) adenosine deaminase. (**c**) 2D ligand interactions of the co-crystallized ligand (CCL), catechin, epicatechin, and pheophytin with (**E**) arginase 2 and (**F**) steroidogenic cytochrome P450 17A1.

The interactions of these ligands with key amino acids at the active site of each receptor was also recorded and are presented in Table 1 above and Figure 4 below. Figure 4a depicts the docking validation, where the co-crystallized ligands were extracted and redocked to the active site of the proteins. The docked pose is superimposed with that of the co-crystallized state to confirm that the co-crystallized ligands could reenact their poses, and the root mean square deviations (RMSDs) were recorded. This was reported for only 2H42 and 3IW4 (as shown in Figure 4a), because the work thenceforth focused on these two receptors, mainly 2H42. Figure 4b shows the 2D ligand interactions of the co-crystallized ligand (CCL), catechin, epicatechin, and pheophytin with human PDE5, protein kinase C alpha, OGT, and adenosine deaminase, while Figure 4c shows the 2D ligand interactions of the co-crystallized ligand (CCL), catechin, epicatechin, and pheophytin with arginase 2 and steroidogenic cytochrome P450 17A1. It was observed that these hit compounds exhibited similar interaction to the amino acids at the active sites, just like the co-crystallized ligands upon binding to the receptors. For instance, on the PDE5 enzyme, catechin and epicatechin exhibited hydrogen bonds with GLN817 and pi-stacking with TYR612 and PHE820, respectively, just like the co-crystallized ligand sildenafil. In addition to these, catechin exhibited an additional H-bond with GLN775 and epicatechin with ASN661 and THR723. Also, on the O-GlcNAc transferase enzyme, epicatechin displayed similar interactions with key amino acids to the co-crystallized ligand. For example, it exhibited an H-bond with both ALA896 and LYS898, just like the co-crystallized ligand, and displayed pi-stacking with HIE901. They also exhibited similar hydrophobic interaction, as shown in Figure 4. These compounds did not display similar interactions to the co-crystallized ligand at 6CIZ; therefore, we focused on the promising targets thenceforth.

**Figure 5:**
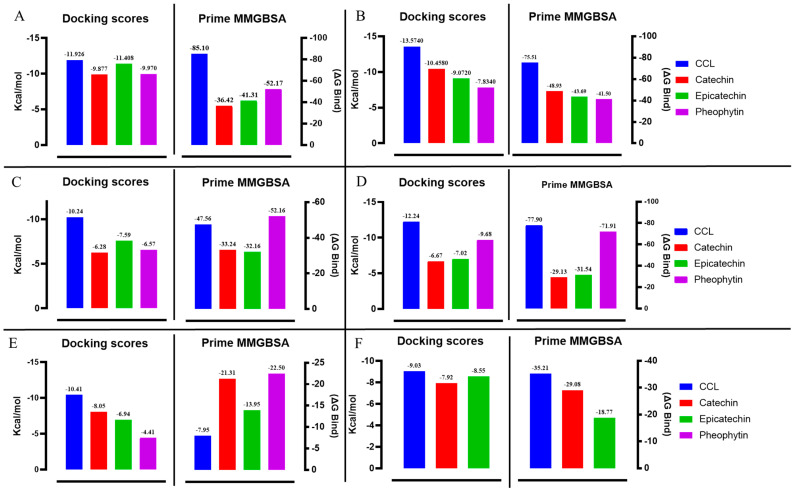
Docking and Prime MM/GBSA scores of the co-crystallized ligand (CCL), catechin, epicatechin, and pheophytin with (**A**) human PDE5, (**B**) protein kinase C alpha, (**C**) OGT, (**D**) adenosine deaminase, (**E**) arginase 2, and (**F**) steroidogenic cytochrome P450 17A1.

The molecular mechanics general Born surface (MM/GBSA) offers an effective means of calculating ligand–protein binding free energy. It is a measure of the amount of free energy involved in a particular set of interactions. For this study, MM/GBSA calculations were conducted using the Prime module of the Schrödinger suite. Our hit compounds showed comparable binding energy to the co-crystallized ligands. For instance, pheophytin showed similar binding energy to the adenosine deaminase enzyme as the co-crystallized ligand, with values of −71.91 and −77.9 Kcal/mol, respectively. Catechin exhibited superior binding energy to the arginase 2 enzyme compared with the co-crystallized ligand (−21.31 vs. −7.95 Kcal/mol). However, the co-crystallized ligands had better binding energies with PDE5 and protein kinase C alpha, as shown in Table 1 and Figure 5.

**Figure 6:**
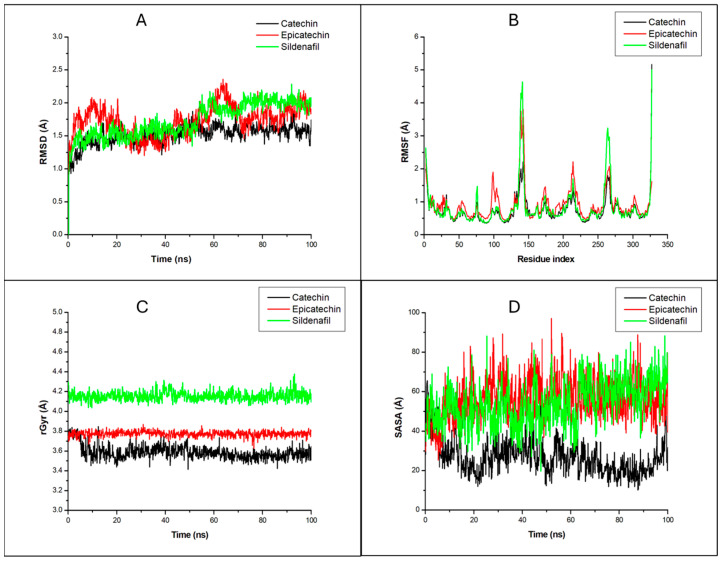
MD simulation results of 2H42 complexed to catechin, epicatechin, and sildenafil. (**A**) RMSD, (**B**) RMSF, (**C**) rGyr, and (**D**) SASA graphical plot. All simulations were carried out using Schrödinger’s Maestro suite (Desmond).

The two compounds (epicatechin and catechin) with the best interactions with the human PDE5 receptor were used for the molecular dynamic study (Figure 6). The root mean square deviation (RMSD), root mean square fluctuation (RMSF), radius of gyration (rGyr), molecular surface area (MolSA), solvent-accessibility surface area (SASA), and polar surface area (PSA), among other parameters, were determined to predict the stability and interraction of these compounds at the receptor’s active site. The results are presented as means ± SEM in Armstrong units (Å), as shown in Table 2. While the RMSD, RMSF, and rGyr give information about protein–ligand stability, SASA gives information about the surface area of the molecule that is accessible by water, and PSA gives information about the solvent-accessible surface area in a molecule that is contributed by nitrogen and oxygen atoms only. Catechin was found to have a lower RMSF, RMSD, and radius of gyration (0.74, 1.517, and 3.58 vs. 0.83, 1.73, and 4.15 respectively) in comparison with sildenafil, while Epicatechin had a comparable RMSD and SASA (1.729 and 56.81 vs. 1.73 and 54.20) when compared with sildenafil, as shown in Table 2.

The MM/GBSA calculations based on the poses from the molecular dynamics for the phosphodiesterase 5 complexes were performed to provide a more comprehensive assessment of the binding energies. The results showed that the co-crystallized ligand, sildenafil, had higher binding energy (MM/GBSA) than catechin and epicatechin (Table 3). This result agrees with that observed with the docked poses.

The absorption, distribution, metabolism, and excretion profile of a compound helps in understanding the physicochemical, pharmacokinetic, and toxicological properties of the compound. For this purpose, the SwissADME web server was used. Catechin and epicatechin (shown in Figure 6) had the same profile save for their consensus log P value, with a slight difference (0.83 vs. 0.85). Aside from that, all other values were the same. It was observed that these compounds had favorable profiles as they did not violate Lipinski’s rule and had a favorable molecular weight, which yielded them favorable synthetic accessibility scores. They also possessed a favorable/acceptable topological polar surface area (TPSA) and consensus log P (except pheophytin), making them promising drug candidates (drug likeness), as shown in Table 4. Regarding their pharmacokinetic properties, bioavailability, and cytochrome P450-metabolizing enzyme-inhibitory potentials, the compounds were highly water-soluble and possessed a high gastrointestinal absorption profile. They did not permeate the blood–brain barrier, had a favorable bioavailability profile, and did not inhibit the CYP1A2, CYP2C19, CYP2C9, CYP2D6, or CYP3A4 enzymes (except gallic acid, which is an inhibitor of CYP3A4). Both catechin and epicatechin are P-glycoprotein substrates, while gallic acid is not, as shown in Table 5.

Synthetic accessibility and bioavailability scores ranged from 1 (very easy to synthesize) to 10 (very difficult to synthesize) and 0 (not bioavailable) to 1 (100% bioavailable). For synthetic accessibility scores, according to the scale, the lower the value, the easier it is to be synthesized; therefore, a lower value is desirable. For bioavailability scores, usually, any compound with a bioavailability score of ≥0.55 is considered ideal and absorbed very well by the body. Therefore, these compounds (except pheophytin) had favorable/acceptable pharmacokinetic and drug-likeness profiles. Overall, pheophytin did not exhibit a favorable or desirable ADME profile and therefore was not included in further studies.

## 3. Discussion

Erectile dysfunction (ED) is increasingly a social problem that affects not only the elderly but men of all ages and significantly affects their quality of life and that of their partners [32]. Though phosphodiesterase 5 inhibitors (PDE5is) are available in the market for the management of ED, there is a need for further development of safer and more effective therapies for this purpose owing to their side effects, including headache and cardiovascular problems [19,33]. There is also a need to explore other plausible targets that may contribute to the initiation and sustenance of penile erection [34,35]. Natural sources (natural products) have contributed to drug development and offer a safe source of lead compounds for drug design, discovery, and development [15,36]. Medicinal plants have been known to contribute to the treatment of various diseases, including erectile dysfunction. Several medicinal plant extracts have shown promising potential in the management of ED, including ginseng, *Garcinia kola*, *Lepidium meyenii*, *Pinus pinaster*, *Tribulus terrestris*, and saffron, among others [16,19], thus making this path of investigation a plausible one. In silico studies, which involve computer-based simulations and molecular modeling, have become powerful tools for predicting the binding affinity of compounds to specific proteins, and are encouraged at the onset of the research process to identify lead compounds from a large library of compounds/database. They are a fast, cost-effective, and integrative means of predicting pharmacological outcomes and improving the drug discovery process.

To understand the interactions between the phytochemicals from *Detarium senegalense* and key enzymes involved in the pathogenesis of ED, molecular docking simulations were conducted, with sildenafil citrate used as the standard. The results showed that catechin and epicatechin had favorable binding energies with most of the selected targets, especially PDE5, protein kinase C alpha, and steroidogenic cytochrome P450 17A1, indicating potential interactions with these enzymes (Table 1). Regarding the PDE5 enzyme, catechin and epicatechin exhibited hydrogen bonds with GLN817 and pi-stacking with TYR612, just like the standard drug, sildenafil citrate. In addition to this, catechin exhibited an additional H-bond with GLN775 and epicatechin with ASN661 and THR723. These interactions (coupled with binding energy, as shown in Table 1) suggests its inhibitory potential on the PDE enzyme, which is responsible for the degradation of cGMP. This finding is in tandem with that reported by [19,21] that flavonoids from *Anonna senegalensis* and *Garcinia kola* inhibit the PDE5 enzyme.

Overactivity of protein kinase C has been shown to contribute to the pathophysiology of ED in diabetes [37]. The inhibition of this enzyme is known to relieve NO/cGMP pathway impairment in penile vascular tissues, thus alleviating ED symptoms [37,38]. Catechin shows strong binding affinity with PKC, with a binding score of −10.46 kcal/mol compared to the −13.57 kcal/mol of the co-crystallized ligand (Table 1 and Figure 5). This shows that catechin has the potential to bind to this target strongly and inhibit its activity/overactivity in ED, thereby aiding sustained erection for satisfactory intercourse via modulation of the NO/cGMP pathway.

In addition, gallic acid, a phenolic compound isolated from this plant [39], also exhibits good binding afinity with PDE5 and PKC, as shown in Figure 1. This further supports the report by [40] that phenolics from natural sources exhibit potential to inhibit key ED enzymes. Also, [41] reported the potential of gallic acid in the management of ED.

The interaction of amino acid residues of the PDE5, PKC, and steroidogenic cytochrome P450 17A1 enzymes with their respective standards and catechin were similar, making these worthy of further investigation. Therefore, molecular dynamic (MD) simulation analysis was performed to evaluate structural and functional relationships with the protein–ligand complex. The MD simulation emulates the biological system and gives information about the stability of the complex, conformational changes, and the individual residue flexibility/fluctuation during simulation [42,43]. The root mean square deviation (RMSD), root mean square fluctuation (RMSF), radius of gyration (rGyr), pressure swing adsorption (PSA), and solvent-accessibility surface area (SASA) were analyzed, as shown in Table 2 (means ± SEM) and Figure 6.

The root mean square deviation (RMSD) measures the degree of structural/conformational variation in a ligand–protein complex over time [43]. A constant and low ligand–RMSD value shows that the ligand maintains a similar pose to its docking/starting pose during the simulation, while a fluctuating RMSD value indicates that there is frequent alteration in the ligand pose in the binding pocket [44]. From our results (Table 2, Figure 6), it was observed that our compounds (epicatechin and catechin) possessed low RMSD values (1.729 ± 0.007 and 1.517 ± 0.005, respectively) throughout the 100 ns simulation, indicating their stability at the receptor site. They also possessed a similar RMSD to the standard drug, sildenafil (1.73 ± 0.008). This result supports our findings from the molecular docking study, where the ligands showed very high binding affinity.

RMSF plots indicate which part of the protein constantly moves throughout a 100 ns simulation and reveals the key residues involved in the strongest interactions with a ligand [43,44]. The compounds had similar RMSF to the standard drug, sildenafil. Also, both compounds maintained a low RMSF value at their most important residues (Figure 6). This indicates that there were minimal movements in the binding region, indicating stable ligand binding.

The rGyr gives information about changes in protein compactness and stability, with lower average rGyr values indicating a protein’s compactness and stability and vice versa [42,43,45]. Our compounds of investigation (epicatechin and catechin) possessed a relatively lower rGyr (3.77 ± 0.001 and 3.58 ± 0.002, respectively) compared to the standard drug, sildenafil (4.15 ± 0.001). The plot showed stability throughout the simulation period for epicatechin-2H42, indicating it did not cause any distortion in the structure of the protein. This supports our molecular docking and RMSD findings that epicatechin and catechin may be effective inhibitors of the human phosphodiesterase enzyme.

While the binding affinity of a compound is important for its activity, it is critical to consider the pharmacokinetic profile and drug likeness of the compounds [46,47]. The Lipinski rule of five (RO5) has been postulated in the drug discovery process to help determine the potential of a molecule to become a drug [48]. As postulated, it is important that a molecule does not violate more than one of these RO5 [49]. The standard drug (sildenafil citrate) violated two of these RO5 [19], while catechin did not violate any of the RO5 (Table 4), thus suggesting that it may well be a promising candidate with better activity and fewer side effects than sildenafil. Its high bioavailability, water solubility, and gastrointestinal absorption profile compared to that of sildenafil position it as a viable lead compound. It did not permeate the blood–brain barrier (Table 5); therefore, it is likely to be devoid of the headache side effects observed with sildenafil. Catechin, epicatechin, and gallic acid also had synthetic accessibility scores of 3.5, 3.5, and 1.22, respectively, making them very easy to synthesize, and their molecular weight of 290.27, 290.27, and 170.12 g/mol, respectively, gives room for possible structural modification/optimization to improve their efficacy. However, pheophytin, though having good binding affinity, possessed a poor ADMET profile and was therefore excluded from further studies. It can therefore be said that the epicatechin- and catechin-rich fraction of *Detarium senegalense* could lead to improved sexual performance with fewer side effects expected compared to sildenafil. Ref. [50] reported on the antidiabetic activity of different fractions of *Detarium senegalense* stem bark extracts. Diabetes mellitus has been known to sometimes lead to erectile dysfunction [51,52]. Therefore, it can be said that this plant has the potential to ameliorate diabetes-associated erectile dysfunction. Regarding the toxicity/safety of this plant, an acute toxicity study revealed no significant signs in toxicity in rats up to 5000 mg/kg [53]. The oral LD50 of catechin and epicatechin has been reported to be >10,000 mg/kg in rodent studies [54,55], while that of gallic acid is >5000 mg/kg [56,57]. This suggests a favorable safety profile of this plant and these compounds.

## 4. Materials and Methods

### 4.1. Virtual Screening and Docking Platform

Literature reviews were conducted to retrieve the phytochemicals that have previously been characterized from *Detarium senegalense* [26,29,50]. These compounds were collected from the PubChem online database (https://pubchem.ncbi.nlm.nih.gov/, accessed on 11 September 2024) and docked to the active sites of the selected targets to predict compounds with the best inhibitory potential to block these targets that have been implicated in erectile dysfunction. Schrödinger Maestro 11.5 was used for the docking study using the standard molecular docking principles, while the SwissADME tool was used for the pharmacokinetic and physicochemical properties, and toxicity prediction.

### 4.2. Phytochemical Library Generation and Preparation

A library of phytochemicals that have previously been characterized from *Detarium senegalense* and reported in the literature was created [26,29,50]. The two-dimensional (2D) structures of these phytochemicals (in SDF format) were retrieved from the PubChem online database (https://pubchem.ncbi.nlm.nih.gov/). The 2D structures were transformed into 3D structures using the ligprep tool in Schrödinger by adding hydrogen atoms, ionizing at pH (7.2 ± 0.2), and removing salt using Ep2i/UNEP/-Zk. The OPLS3e force field was utilized for ionization and tautomeric state formation, as previously described [58].

### 4.3. Receptor Retrieval and Preparation

The three-dimensional (3D) X-ray crystal structure of the selected receptors, human phosphodiesterase 5 (PDB ID: 2H42) [59], arginase 2 (PDB ID: 4I06) [60], steroidogenic cytochrome P450 17A1 (PDB ID: 6CIZ) [61], protein kinase C alpha (PDB ID: 3IW4) [62], O-GlcNAc transferase (PDB ID: 6MA5) [63], and adenosine deaminase (PDB ID: 1NDZ) [64], were retrieved from the Protein Data Bank (https://www.rcsb.org), with their corresponding bound ligands. The PyMOL molecular graphics system (version 2.5, Schrödinger, LLC., New York, NY, USA) was used for visualization of the proteins. The protein preparation wizard tool in Maestro was used to prepare the protein before performing the molecular docking, as previously described [58]. Briefly, bond order assignment was conducted, hydrogens added, zero-order metal bonds made, disulfide bonds created, water molecules removed, and het states generated using Epik at pH 7.0 ± 0.2 during protein production. Protein refinement was performed by optimizing the H-bond assignment, and then the OPLS3e force field was used to minimize the protein.

### 4.4. Receptor Grid Generation

The Receptor Grid Generation tool was used to create the prepared protein grid on the binding site (Glide Grid). The receptor grid depicts the area where the ligand and protein interact. The coordinate of the co-crystallized ligand was used to specify and generate the receptor grid/active site for docking. By selecting the co-crystallized ligand at the active site of the receptor, the binding location was automatically mapped (by a cubic grid box), covering all of the amino acid residues at the active site. The default Glide Grid setting was used, and the produced grid’s three-dimensional coordinates X, Y, and Z, respectively, for each of the proteins were 2H42: (12.42, −3.89, 2.05) Å, 3IW4: (5.47, 29.95, 52.13) Å, 6CIZ: (29.4, 142.65, 40.05) Å, 6MA5: (−0.23, −45.62, 15.45) Å, 1NDZ: (48.6, 52.65, 19.22) Å, 4I06: (34.04, 85.71, 72.04) Å respectively.

### 4.5. Molecular Docking

Docking was achieved on Maestro 11.5 with the Glide tool using extra-precision (XP) docking techniques. The default setting of the Glide tool was used, with the ligand sampling set to be flexible, no constraints set, and post-docking minimization enabled. The co-crystallized ligands were extracted and re-docked into the active site to validate the molecular docking study.

### 4.6. Molecular Mechanics/Generalized Born Surface Area (MM/GBSA)

The potential binding free energy of the receptor–ligand docked complexes was calculated using Prime MM/GBSA in the Schrödinger suite. For the free binding energy calculation of the docked complexes, the solvent model and force field were set to VSGB and OPLS3, respectively, while other options were left at the default settings, as previously reported [58].

### 4.7. Molecular Dynamic (MD) Simulation and Trajectory Analysis

The molecular dynamic (MD) simulation for the native phosphodiesterase 5 enzyme (2H42) and the two complexes (2H42-epicatechin and 2H42-catechin) was executed using the Desmond module of the Schrödinger suite. The system setup, MD production, and trajectory analysis were performed as previously reported [65]. All simulations were carried out using the OPLS2005 force field. The protein–ligand complexes were bound in an orthorhombic box, with the box size calculation method set as buffer, all three distances set at 10 Å, then the volume of the box minimized. The TIP3P water model was used as the solvent model. Sodium and chloride ions were added to neutralize the overall charge of the system, and the salt concentration was set to 0.15 M to mimic physiological conditions. The standard protocols within the Maestro environment were employed to initially prepare and minimize the system. System relaxation was undertaken in an NPT ensemble at 300 K and 1 atm using a Nosé–Hoover thermostat and a Martyna–Tobias–Klein barostat, respectively. The MD simulation was performed for 100 nanoseconds (ns), and the trajectory sampling was set at an interval of 100 ps with 1000 frame numbers, allowing for extensive sampling of the conformational space. During the MD simulation, the long-range electrostatic interactions were calculated using the particle mesh Ewald (PME) method. The outputs of the simulation were visuallized and analyzed by MS-MD trajectory analysis and a simulation interaction diagram. The data were plotted using Origin version 6.0.

### 4.8. Absorption, Distribution, Metabolism, Excretion, and Toxicological (ADMET) Prediction

The SwissADME online server (http://www.swissadme.ch/index.php#, accessed on 25 May 2024) was used to estimate the physicochemical, pharmacokinetic, and toxicological properties of the lead compounds, which predict their ADMET profile in the human body [66].

## 5. Conclusions

Based on the analysis of the molecular docking results, this study revealed that some phytochemicals from *Detarium senegalense* possess a favorable binding affinity with human PDE5, protein kinase C alpha, and steroidogenic cytochrome P450 17A1. The docking analysis showed that catechin, epicatechin, and gallic acid possess a high affinity for these receptors. Catechin stands out from all these phytochemicals because of its superior binding energies, favorable pharmacokinetic profile, and good binding conformation/interaction at the receptor site. Our results therefore suggest that catechin can act as a multi-target inhibitor of key enzymes involved in the pathogenesis of erectile dysfunction. Therefore, this study suggests that the flavonoid-rich fraction of *Detarium senegalense* could lead to improved sexual performance and sustained erection for satisfactory sexual intercourse. Also, catechin could serve as a potential lead compound for development of a drug for the management of ED, owing to its satisfactory pharmacokinetic profile, non-violation of the Lipinski rule of five, good binding affinity, and good interaction at the receptor sites of the enzymes. It is important to note that while in silico studies provide valuable insights, experimental validation is necessary to confirm the inhibitory activity of these compounds. Also, there has been no report on the quantitative analysis of these compounds (to determine the amount of catechin and epicatechin) in *Detarium senegalense*. Therefore, there is a need to quantify these compounds that are found in the plant to support their role in the management of ED. Moreover, further research is needed to optimize their pharmacological properties and evaluate their effectiveness in animal models and clinical trials. If successful, these compounds could open new avenues for the development of cost-effective and safe drugs for the management of erectile dysfunction.

Overall, this computational investigation supports the potential of *Detarium senegalense* phytochemicals as promising candidates for developing new, safer, and more effective therapies for erectile dysfunction, offering an alternative to current treatments with fewer side effects.

## Figures and Tables

**Table 1:** Showing the Glide scores (kcal/mol), MM/GBSA (kcal/mol), and interacting residues of each target with their co-crystallized ligands and best hit compounds.

Target	Compound	Glide Score	MM/GBSA	Key Interaction
2H42	CCL	−11.926	−85.1	H-bond-GLN817 Pi-stacking-TYR612, PHE820
	Catechin	−9.877	−36.42	H-bond-GLN775, GLN817 Pi-stacking-TYR612, HIS613
	Epicatechin	−11.408	−41.31	H-bond-ASN661, THR723, GLN817 Pi-stacking-HIS613, PHE820
	Pheophytin	−9.97	−52.17	H-bond-ASN662, THR723 Pi-stacking-PHE820
3IW4	CCL	−13.574	−75.51	H-bond-THR401, GLU418, VAL420, ASP467 Pi-cation-ASP424, ASP467
	Catechin	−10.458	−48.93	H-bond-GLU387, VAL420
	Epicatechin	−9.072	−43.69	H-bond-GLU418, ASP467, ASN468
	Pheophytin	−7.834	−41.5	H-bond-GLU418, GLY423 Pi-stacking-PHE350
6MA5	CCL	−10.236	−47.56	H-bond-GLN839, ALA896, LYS898 Pi-stacking-HIE558, HIE901 Salt bridge-LYS842
	Catechin	−6.276	−33.24	H-bond-ASN557, PHE837, ALA896
	Epicatechin	−7.586	−32.16	H-bond-HIE562, ALA896, LYS898 Pi-stacking-HIE901
	Pheophytin	−6.573	−52.16	H-bond-HIE558, PHE837, GLN839, HIE920, THR922
1NDZ	CCL	−12.244	−77.9	H-bond-HIS17, ASP19, GLU217, ASH296 Pi-stacking-HIS17, PHE61, PHE65
	Catechin	−6.667	−29.13	H-bond-GLY184, GLU217, ASH296
	Epicatechin	−7.019	−31.54	H-bond-LEU56, GLU217
	Pheophytin	−9.682	−71.91	H-bond-HIS17, ASP19, SER103, ASP185, GLU217
4IO6	CCL	−10.414	−7.95	H-bond-ASP147, SER156, ASN158, HIS160, ASP202, GLU205
	Catechin	−8.048	−21.31	H-bond-HIS160, GLY161, ASP202, GLU205, ASP253 Pi-stacking-HIE145, HIS160
	Epicatechin	−6.935	−13.95	H-bond-THR154, ASN158, GLY161, ASP200, GLU296 Pi-stacking-HIE145, HIS160
	Pheophytin	−4.408	−22.5	H-bond-SER155, ASP200, VAL201, ASP202, GLU205
6CIZ	CCL	−9.029	−35.21	H-bond-ASN202
	Catechin	−7.921	−29.08	H-bond-ASP298, VAL482
	Epicatechin	−8.547	−18.77	H-bond-ASP298, VAL482

**Table 2:** Interactive properties of MDs of the PDE5 receptors and protein–ligand interactions.

Receptor	Ligand	P_RMSF	RMSD	rGyr	MolSA	SASA	PSA
2H42	Sildenafil	0.83 ± 0.035	1.73 ± 0.008	4.15 ± 0.001	410.0 ± 0.17	54.20 ± 0.34	140.8 ± 0.12
	Epicatechin	0.91 ± 0.027	1.729 ± 0.007	3.77 ± 0.001	255.7 ± 0.04	56.81 ± 0.33	245.8 ± 0.08
	Catechin	0.74 ± 0.023	1.517 ± 0.005	3.58 ± 0.002	253.5 ± 0.05	26.88 ± 0.27	259.8 ± 0.11

Note: Values are presented as means ± standard error of mean (SEM) measured in Armstrong units (Å). RMSD: complex root mean square deviation; P_RMSF: protein root mean square fluctuation; rGyr: radius of gyration; MolSA: molecular surface area; SASA: solvent-accessibility surface area; PSA: polar surface area.

**Table 3:** Calculated MM/GBSA values of the MD trajectories of the PDE5 protein–ligand complex poses.

Receptor	Ligand	MM/GBSA
2H42	Sildenafil	−76.29 ± 0.38
	Epicatechin	−38.84 ± 0.69
	Catechin	−45.72 ± 0.46

Note: Values are presented as mean ± standard error of mean (SEM) measured in kcal/mol.

**Table 4:** In-silico drug-likeness prediction of the catechin and gallic acid.

Compound	MW	#H-Bond Acceptors	#H-Bond Donors	TPSA	Consensus Log P	#Lipinski Violation	Synthetic Accessibility
Catechin	290.27	6	5	110.38	0.83	0	3.5
Gallic acid	170.12	5	4	97.99	0.21	0	1.22
Pheophytin	871.2	8	2	121.94	9.91	2	10

**Table 5:** The pharmacokinetic properties, bioavailability, and cytochrome P450-metabolizing enzyme-inhibitory potentials of the compounds.

	GI Absorption	BBB Permeant	P-Glycoprotein Substrate	CYP1A2 Inhibitor	CYP2C19 Inhibitor	CYP2C9 Inhibitor	CYP2D6 Inhibitor	CYP3A4 Inhibitor	Bioavailability Score
Catechin	High	No	Yes	No	No	No	No	No	0.55
Gallic acid	High	No	No	No	No	No	No	Yes	0.56
Pheophytin	Low	No	Yes	No	No	No	No	No	0.17

## Data Availability

The data presented in this study are available upon request from the corresponding author.
